# Supplementary material for: WordCommentsAnalyzer: A windows software tool for qualitative research
Source: F1000Res. 2018 Sep 4;7:536. Originally published 2018 May 3. [Version 2] doi: 10.12688/f1000research.14819.2 (PMC6137411; doi:10.12688/f1000research.14819.2)
Supplement: Supplementary file 3 [file f1000research-7-17614-s0002.tgz › b2a79496-4ada-47c4-9682-a99ce867836d.zip!/Eight Iranian Health Journals/Archives of Rehabilitation_30.docx]

© Archives of Rehabilitation. This work is licensed under CC BY-NC 4.0 (https://creativecommons.org/licenses/by-nc/4.0/).

Volume & Issue: دوره 12 - شماره 4

Title: محاسبه پایداری دینامیک راه رفتن در افراد با ضایعه لیگامان صلیبی قدامی برای ارزیابی تأثیر تمرینات اغتشاشی

http://rehabilitationj.uswr.ac.ir/article-1-415-fa.html

هدف: در این تحقیق با استفاده از روش دینامیک غیرخطی، از شاخص ثبات دینامیک برای بررسی تأثیر اغتشاش درمانی در افراد مبتلا به ضایعه لیگامان صلیبی قدامی استفاده شده است. روش بررسی: نمونه‌گیری به صورت غیراحتمالی قضاوتی از بین مردان ورزشکاری که حداقل 6 ماه از ضایعه لیگامان صلیبی قدامی آنها گذشته بود صورت گرفت. با استفاده از تخته‌های تعادل، 10 جلسه اغتشاش درمانی انجام گرفت. داده برداری سینماتیک مفاصل اندام‌ تحتانی با استفاده از الکتروگونیامتر قبل و بعد از درمان انجام شد. داده‌های سینماتیک مربوط به مفصل زانو در 60 سیکل راه‌رفتن برای محاسبه شاخص ثبات دینامیک مورد استفاده قرارگرفت. سری‌های زمانی در پنج بعد بازسازی شدند و نمای لیاپانوف زمان محدود برای 7 نفر قبل و بعد از دریافت تمرینات اغتشاشی محاسبه شد. از آزمون ناپارامتری ویلکاکسون برای بررسی تأثیر تمرینات استفاده شد. یافته‌ها: مقدار شاخص ثبات دینامیک قبل و بعد از تمرینات به ترتیب ۰/۲±۰/۷ و ۰/۲±۰/۵ بود. تحلیل آماری نشان داد شاخص ثبات دینامیک راه‌رفتن افراد مبتلا به آسیب لیگامان صلیبی قدامی بعد از تمرینات اغتشاشی بهبود معناداری داشته است (۰/۰16=P). نتیجه‌گیری: تمرینات اغتشاشی در بهبود تعادل دینامیک افراد مبتلا به آسیب لیگامان صلیبی قدامی حین راه رفتن مؤثر بوده است. بنابراین با استفاده از محاسبات دینامیک غیرخطی می‌توان بستر نظری استواری برای طراحی و ارزیابی درمان‌های توانبخشی اختصاصی ضایعه لیگامان صلیبی قدامی ایجاد نمود. این روش‌های محاسباتی می‌تواند برای ارزیابی عملکردی دیگر مداخلاتی که در حرکاتی مثل راه رفتن تأثیر می‌گذارند نیز استفاده شود.

Title: تجارب و دیدگاه‌های کاردرمان‌گران از حرفه کاردرمانی

http://rehabilitationj.uswr.ac.ir/article-1-725-fa.html

هدف: توسعه و ارتقا هر حرفه‌ای، ارتباط مستقیم با شناخت تجارب و دیدگاههای افراد شاغل در آن حرفه دارد. این تحقیق در راستای شناخت دیدگاه‌ها و تجارب شغلی، آموزشی و بالینی شاغلین رشته حرفه کاردرمانی انجام گردیده است. روش بررسی: این مطالعه با رویکرد کیفی از نوع تحلیل محتوا است. شرکت‌کنندگان آن 15 کاردرمان‌گر شاغل در کلینیک‌های سطح شهر تهران در سال 1387 بودند. روش جمع آوری داده‌ها استفاده از مصاحبه عمیق نیمه ساختار یافته بود. جهت تحلیل داده‌ها از روش تحلیل محتوای کیفی و به صورت مقایسه‌ای مداوم استفاده شد. یافته‌ها: در آنالیز اولیه ۱۷۰ کد اولیه بدست آمد که در ۱۵ زیر مضمون و ۵ مضمون اصلی یا درونمایه از بین آنها شامل: «عدم شناخت متقابل جامعه و کاردرمان‌گران از یکدیگر و نگرش فرهنگی-اجتماعی مبهم»، «عدم رضایتمندی از حرفه»، «داشتن نگاهی نو و خلاقانه به حرفه کاردرمانی»، «گستردگی و بین حرفه‌ای بودن ماهیت درونی رشته» و «سیستم آموزشی نامتناسب، غیریکپارچه و غیر محرک» استخراج شد. تجارب کاردرمان‌گران نشان داد که شاغلین این حرفه ضمن مهم و سودمند دانستن حرفه خود، از هم گسیختگی، نامتناسب بودن، و گسترده بودن آموزش‌ها و شرح وظایف، عدم شناخت مردم از حرفه، آزردگی خاطر دارند؛ و همچنین تجارب آنها تحت تأثیر شدید عکس العمل و نگاه اطرافیان و اساتید نسبت به آنها دارد. به طوری که اگر نگاه اساتید و همکاران مثبت باشد، دانشجویان امیدوارتر نسبت به رشته خود بوده و نسبت به آن افتخار می‌کنند و در نهایت خواهان حمایت مسئولین و سازمانهای مرتبط هستند. نتیجه‌گیری: تجارب و دیدگاه کاردرمان‌گران در رابطه با حرفه‌شان مثبت بوده و متأثر از نگرش دیگران، شامل: اساتید، همکاران حرفه‌ای و مردم می‌باشد. تجارب آنها نشانگر عدم محتوای آموزشی متناسب با نیازهای جامعه و فرهنگ مردم ایران بوده و بین آموزش و بالین از نظر آنها شکاف وجود دارد، آنها خواستار افزایش آگاهی جامعه از حرفه‌شان و حمایت مسئولین می‌باشند.

Title: مقایسه تأثیر گوه خارجی خلف پا و گوه خارجی قدام پا بر گشتاور اداکشن زانو در دانشجویان پسر سالم

http://rehabilitationj.uswr.ac.ir/article-1-659-fa.html

هدف: کفی‌های دارای گوه خارجی برای کاهش نیروی عمل کننده روی کمپارتمان داخلی زانو طراحی شده‌اند.هدف این مطالعه بررسی اثرش محل قرارگیری گوه خارجی در زیر سطح کف پایی (در قسمت خارجی خلف و خارجی قدام پا) بر گشتاور اداکشن زانو و نیروهای عکس العمل زمین بود. روش بررسی: در این مطالعه پیش‌آزمون-پس‌آزمون، تجزیه و تحلیل سه بُعدی الگوی راه رفتن روی 20 مرد سالم بین 30-18 انجام شد. گشتاور اداکشن زانو و نیروهای عکس العمل زمین بین سه وضعیت کفی ساده، کفی با گوه خارجی خلف پا با زاویه 6 درجه و کفی با گوه خارجی قدام پا با زاویه 66 درجه با هم مقایسه شدند. داده‌ها با استفاده از آزمون تی زوجی تحلیل شد. یافته‌ها: هیچ تفاوتی در گشتاور اداکشن زانو در وضعیت استفاده از کفی ساده (9/72±1/501) و کفی‌های دارای گوه خلفی خارجی (9/866±2/141) و قدامی خارجی (9/952±1/986) مشاهده نشد (P>0/05).  نتیجه‌گیری: بر پایه یافته‌های مطالعه حاضر، جایگاه قرار گیری گوه خارجی زیر سطح کف پایی، چه در قسمت پاشنه و چه در قسمت جلو پا تأثیری بر اثرگذاری این نوع کفی‌ها بر روی گشتاور اداکشن زانو و نیروهای عکس العمل زمین نداشت.

Title: بررسی تکرارپذیری آزمون کیفیت حرکات اندام فوقانی در کودکان فلج مغزی

http://rehabilitationj.uswr.ac.ir/article-1-897-fa.html

هدف: هدف این پژوهش، بررسی تکرار پذیری اینتر ریتر، اینترا ریتر با و بدون فیلمبرداری آزمون کیفیت حرکات اندام فوقانی در کودکان فلج مغزی اسپاستیک بود. روش بررسی: در یک مطالعه مقطعی به اعتبار سنجی پرسشنامه کیفیت مهارت های اندام فوقانی در کودکان فلج مغزی پرداخته شد. ۵۰ کودک فلج مغزی نیمه بدن ۹۵-۱۹ ماهه با میانگین سنی ۲۵/۷±۶۱/۳۱ ماه با انتخاب غیر تصادفی در دسترس در مطالعه شرکت کردند. پس از کسب رضایت از والدین ارزیابی اینترا ریتر در یک جلسه انجام و ارزیابی اینتر ریتر ۱۰ روز بعد همراه با فیلمبرداری انجام شد. سپس آزمونگر سوم با مشاهده فیلم ۴۶ کودک از ۵۰ کودک، ارزیابی مجدد نمود. از آزمون ضریب همبستگی اسپیرمن (ICC) برای تکرارپذیری اینتر ریتر، اینترا ریتر با و بدون فیلمبرداری و سیستم طبقه بندی عملکردی حرکات درشت۶۶ جهت تعیین عملکرد کودک استفاده گردید. یافته‌ها: همبستگی اینتراریتر (۰/۹۹۶-۰/۷۷۴) و همبستگی اینترریتر بدون فیلمبرداری (۰/۹۹۸-۰/۶۶۳) و با فیلمبرداری بین آزمونگران اول و سوم(۰/۹۷۴-۰/۷۱۰) و دوم و سوم (۰/۹۳۸-۰/۶۵۲) در خرده مقیاس ها و نمره کلی پرسشنامه با مقدار احتمال (۰/۰۱< P) بود.  نتیجه گیری: تکرار‌پذیری اینترا‌ریتر و اینتر‌ریتر با و بدون فیلمبرداری آزمون کیفیت مهارت های اندام فوقانی در کودکان فلج مغزی بالا است و بنابراین به عنوان یک آزمون معتبر برای ارزیابی کیفیت حرکات اندام فوقانی این کودکان پیشنهاد می شود.

Title: مقایسه تأثیر پیچیدگی رمزگذاری واجی بر روانی گفتار کودکان عادی و لکنتی ۹-۶ سال شهر تهران

http://rehabilitationj.uswr.ac.ir/article-1-404-fa.html

هدف: لکنت یکی از شایع‌ترین اختلالات گفتاری است با وجود این، علت آن به درستی مشخص نیست و احتمالاً یک اختلال چند عاملی است. هدف از تحقیق حاضر بررسی و مقایسه تأثیر پیچیدگی رمز گذاری واجی بر روانی گفتار کودکان عادی و لکنتی ۹-۶ سال شهر تهران در سال 1388 می‌باشد. روش بررسی: این پژوهش مقطعی و توصیفی تحلیلی بر روی 18 کودک مبتلا به لکنت شدید و خیلی شدید و 18 کودک عادی انجام گرفت. روش نمونه گیری در مورد کودکان لکنتی از نوع در دسترس و گروه عادی بر اساس همتا سازی با کودکان لکنتی از نظر سن و جنس و محل سکونت بود. برای بررسی عملکرد فرآیند رمز گذاری واجی و تأثیر پیچیدگی این فرآیند بر روانی گفتار از آزمون تکرار ناکلمه استفاده شد. محرک‌ها به طور تصادفی و با فاصله زمانی 5 ثانیه از طریق یک هدفون مدل (SOMIC SM-818) متصل به کامپیوتر برای هر آزمودنی پخش می‌شدند سپس از وی درخواست می‌شد در این فرصت محرک شنیده شده را تکرار کند. یافته‌ها: نتایج پژوهش نشان دادند که در کودکان مبتلا به لکنت درصد روانی گفتار، با افزایش پیچیدگی واجی کل محرک‌ها، به طور معناداری بیشتر از کودکان عادی کاهش یافت (۰/05>P). نتیجه‌گیری: نتایج نشان داد که در کودکان مبتلا به لکنت، رمز گذاری واجی به خوبی انجام نمی‌شود و با پیچیدگی واجی این نقص بیشتر می‌شود و این امر بر اساس فرضیه اصلاح پنهان می‌تواند سبب ایجاد خود اصلاحی پنهان و به دنبال آن، وقوع انواع الگوهای لکنت شود.

Title: رابطه شیوه‌های فرزندپروری والدین، خودپنداره و اضطراب مدرسه در دانش آموزان پسر عقب مانده ذهنی خفیف 12 تا 18 ساله شهر کرمانشاه

http://rehabilitationj.uswr.ac.ir/article-1-657-fa.html

هدف: هدف این مطالعه تعیین رابطه شیوه‌های فرزندپروری والدین و اضطراب مدرسه با توجه به متغیر خودپنداره در دانش آموزان پسر عقب مانده ذهنی خفیف 18-12 سال شهر کرمانشاه بود. روش بررسی: روش نمونه گیری در این مطالعه همبستگی، به صورت تمام شمار بود. جمعیت مورد مطالعه شامل کلیه دانش آموزان پسر عقب مانده ذهنی خفیف 18-12 سال شهر کرمانشاه می‌شد. با توجه به ملاک‌های ورود و خروج آزمون خودپنداره بر روی 125 دانش آموز اجرا شد، که بر اساس نمره آزمون، 83 نفر انتخاب شده و آزمون شیوه‌های فرزندپروری توسط والدین آنها و آزمون اضطراب مدرسه توسط معلمین‌شان تکمیل شد. داده‌های حاصل با آزمون‌های آماری کلموگروف اسمیرنوف، همبستگی پیرسون و اسپیرمن و آزمون تحلیل کوواریانس مورد تجزیه و تحلیل قرار گرفتند. یافته‌ها: شیوه فرزندپروری مقتدرانه پدر (۰/002=P) و مادر (۰/001=P) رابطه منفی معنادار با اضطراب مدرسه داشت؛ همچنین شیوه فرزندپروری مستبدانه پدر (۰/009=P) و مادر (۰/020=P) رابطه مثبت معناداری با اضطراب مدرسه نشان داد. ولی شیوه فرزندپروری سهل گیرانه پدر (۰/319=P) و مادر (۰/452=P) رابطه معناداری با اضطراب مدرسه نداشت و مشخص شد که خودپنداره تأثیر معناداری بر اضطراب مدرسه دانش آموزان عقب مانده ذهنی دارد (۰/004=P). نتیجه‌گیری: نتایج آزمون‌های آماری نشان داد شیوه‌های فرزندپروری والدین بر اضطراب مدرسه دانش آموز اثرگذارند، ولی خودپنداره در تعدیل اثر شیوه‌های فرزندپروری بر اضطراب مدرسه نقش مهمی دارد. به این معنا که دانش آموزانی که خودپنداره قوی دارند کمتر از دانش آموزانی که خودپنداره ضعیف دارند، اضطراب مدرسه را تجربه می‌کنند.

Title: مقایسه اثربخشی آموزش راهبردهای شناختی و شناختی-فراشناختی مبتنی بر مهارت‌های حل مسئله ریاضی در دانش‌آموزان دختر کم‌توان ذهنی پایه سوم راهنمایی

http://rehabilitationj.uswr.ac.ir/article-1-771-fa.html

هدف: هدف این پژوهش مقایسه اثربخشی آموزش راهبردهای شناختی و شناختی- فراشناختی مبتنی بر مهارت‌های حل مسئله ریاضی در دانش‌آموزان دختر کم توان ذهنی پایه سوم راهنمایی در شهرستان‌های تهران است. روش بررسی: در این مطالعه آزمایشی با طرح پیش آزمون- پس آزمون با گروه کنترل، به روش نمونه گیری خوشه‌ای چند مرحله ای از میان شهرستان‌های تهران سه شهرستان قرچک، شهر ری و شهریار انتخاب شدند و از هر شهرستان یک مدرسه و از هر مدرسه به صورت تصادفی 15 دانش آموز دختر کم توان ذهنی پایه سوم راهنمایی انتخاب شدند. پس از انتساب تصادفی دانش‌آموزان در دو گروه آزمایش و یک گروه کنترل، دانش‌آموزان گروه‌های آزمایشی راهبردهای شناختی و شناختی- فراشناختی، طی 15 جلسه تحت آموزش قرار گرفتند و به دانش‌آموزان گروه کنترل آموزش راهبردها ارائه نشد. ابزار جمع آوری اطلاعات، آزمون هوش وکسلر کودکان تجدید نظر شده برای همتا کردن گروه‌ها به لحاظ بهره هوشی، آزمون‌های معلم ساخته حل مساله ریاضی و بسته آموزشی راهبردهای شناختی و شناختی- فراشناختی بود. داده‌های به دست آمده با استفاده از روش‌های آمار توصیفی (میانگین، انحراف معیار و جدول فراوانی) و تحلیل کوواریانس تجزیه و تحلیل شد. یافته‌ها: نتایج نشان داد که میانگین نمرات آزمون حل مساله ریاضی گروه راهبرد شناختی - فراشناختی به طور معناداری بیشتر از گروه شناختی (P<0/005) بود. همچنین اختلاف میانگین گروه شناختی به طور معناداری بیشتر از گروه کنترل (۰/003>P) بود. نتیجه‌گیری: آموزش راهبرد شناختی-فراشناختی و راهبرد شناختی عملکرد دانش‌آموزان را در حل مسأله ریاضی افزایش داد. همچنین، آموزش راهبرد شناختی–فراشناختی در مقایسه با آموزش راهبرد شناختی موجب بهبود عملکرد دانش‌آموزان در حل مساله ریاضی شد.

Title: اختلالات روان‌پزشکی و مشکلات روانی-اجتماعی مراجعه‌کنندگان به مراکز مشاوره سازمان بهزیستی کل کشور

http://rehabilitationj.uswr.ac.ir/article-1-718-fa.html

هدف: برنامه‌ریزی برای ارائه خدمات بهداشت روان به افراد جامعه مستلزم بررسی ویژگی‌های مخاطبان و مشکلات ارجاعی برای ترسیم تصویر روشنی از وضعیت موجود است. تعداد مطالعات انجام شده در این زمینه اندک است. پژوهش حاضر با هدف شناخت ویژگی‌های جمعیت‌شناختی و بررسی وضعیت اختلالات روانپزشکی و مشکلات روانی-اجتماعی مراجعه‌کنندگان به مراکز مشاوره سازمان بهزیستی کل کشور انجام شد. روش بررسی: این پژوهش از نوع مطالعات مقطعی-تحلیلی است. داده‌های پژوهش مبتنی بر اطلاعات موجود در پرونده‌های کل مراجعین به مراکز خدمات مشاوره‌ای سازمان بهزیستی کشور طی سال‌های 1380 تا 1388 بود که قبلاً از طریق مصاحبه نیمه ساختاریافته و پرسشنامه مشخصات روان‌شناختی جمع‌آوری شده بود و تعداد 21766 نمونه با روش نمونه‌گیری منظم انتخاب شدند. برای تحلیل داده‌ها از آزمون مجذور خی، نیکویی برازش و ضریب کاپا در نرم افزار SPSS نسخه 16 استفاده شد. یافته‌ها: یافته‌های پژوهش نشان داد که مخاطبین اصلی خدمات مشاوره عمدتاً از جامعه زنان، افراد متأهل، گروه سنی 40-21 سال و دارای تحصیلات دیپلم و بالاتر تشکیل‌می‌شود. در میان اختلالات روانپزشکی، تشخیص اختلالات خلقی و اضطرابی بیش‌تر از سایر تشخیص‌ها بود. از میان مشکلات روانی–اجتماعی، مشکلات بین فردی بالاترین سهم مراجعین به مراکز مشاوره سازمان بهزیستی را به خود اختصاص داد. نرخ تطابق تشخیص‌های اول و دوم برابر ٪74/4، کاپای 0/27 و معنادار بود (0/001>P). نتیجه‌گیری: با توجه به اینکه بیش‌ترین نرخ شیوع و مراجعات حضوری و تلفنی مراکز مشاوره سازمان بهزیستی به اختلالات خلقی و اضطرابی و مشکلات بین فردی اختصاص دارد، خدمات تخصصی مراکز مشاوره سازمان بهزیستی باید در راستای اولویت دهی به شناسایی و درمان این اختلالات سازمان‌دهی شود.

Title: بررسی تأثیر سطوح زیر و بمی و بلندی صوت بر الگوی انسداد چاکنای در افراد دارای صوت بهنجار

http://rehabilitationj.uswr.ac.ir/article-1-349-fa.html

هدف: الگوی انسداد چاکنای مهم‌ترین نشانه استروبوسکوپی برای قضاوت راجع به بهنجار بودن عملکرد حنجره است. تحقیقات اخیر نشان می‌دهد الگوی انسداد کامل تنها الگوی بهنجار نیست. هدف از این پژوهش بررسی الگوی انسداد در افراد بهنجار و بررسی تأثیر مشخصات آواسازی بر آن می‌باشد. روش بررسی: در این مطالعه توصیفی تحلیلی مقطعی، پس از تاریخچه‌گیری، ارزیابی شنیداری-ادراکی جامع صوت و شاخص معلولیت صوت 218 دانشجو، 80 نفر (58 مؤنث و 22 مذکر) با میانگین سنی ۲۱٫19 به صورت تصادفی انتخاب شدند که ارزیابی‌ها دال بر بهنجار بودن صوت آنها بود. ارزیابی ویدئواستروبوسکوپی حین تولید واکه /I/ در آواسازی عادتی، زیر، بم، بلند و آهسته انجام، و الگوهای انسداد چاکنای در سطوح فوق ثبت گردید. ضمن ارائه آمار توصیفی، الگوهای انسداد چاکنای با استفاده از آزمون غیر پارامتری فیشر در سطوح آواسازی بین افراد مذکر و مؤنث مقایسه گردید. یافته‌ها: در مجموع فراوان‌ترین الگو، انسداد کامل و پس از آن شکاف خلفی Y بود. مقایسه فراوانی الگوها بیانگر تفاوت معنادار بین افراد مذکر و مؤنث (0/001>P) و سطوح عادتی، زیر، بم، بلند و آهسته بود (0/001>P). تفاوت معنادار در الگوی انسداد حین تولید انواع زیر و بمی در افراد مؤنث (0/001>P) و انواع بلندی‌ها در افراد مذکر (0/001>P) دیده شد. نتیجه‌گیری: در ارزیابی ویدئواستروبوسکوپی، مشاهده انسداد ناکامل در افراد بهنجار دور از انتظار نیست. مشخصات آواسازی می‌تواند بر شکل انسداد به طور معنادار تأثیر بگذارد، لذا کلیه سطوح آواسازی باید مورد ارزیابی قرار گیرد، اگرچه نحوه تأثیر در افراد مذکر و مؤنث مشابه نیست.

Title: بررسی و مقایسه اثرات بالشتک حمایتی قوس داخلی و گوه‌های پاشنه و پنجه‌پا بر تغییر میزان نیروی اعمال‌شده بر اکستانسور مکانیزم زانو در فاز ایستایی راه‌رفتن در دختران 25-19 سال دارای کف پای صاف

http://rehabilitationj.uswr.ac.ir/article-1-387-fa.html

هدف: صافی کف پا یکی از اختلالات شایع است که می‌تواند به علت وجود ارتباط بیومکانیکی بین پا و بخش‌های دیگر اندام تحتانی، موجب بروز مشکلاتی در دیگر قسمت‌های اندام‌ تحتانی به خصوص زانو گردد. این تحقیق با هدف مقایسه میزان نیروی وارد بر مکانیزم اکستانسوری زانو در دختران دارای کف پای صاف پس از پوشیدن سه نوع مختلف ارتز (بالشتک حمایتی قوس داخلی، بالشتک حمایتی قوس داخلی به‌علاوه گوه داخلی پاشنه، بالشتک حمایتی قوس داخلی به‌علاوه گوه خارجی پنجه) صورت گرفت. روش بررسی: در این مطالعه شبه تجربی، 10 دانشجوی دختر 19-25 سال دارای کف پای صاف انعطاف‌پذیر به روش نمونه‌گیری در دسترس و بوسیله تست فیس‌لاین انتخاب شدند و پس از پوشیدن هر کدام از انواع ارتزها، نشان‌گرهای منعکس کننده نور بر روی نقاط آناتومیکی مشخص روی پوست فرد چسبانده شد. داده‌های کینماتیک و کینتیکی آنها در حین راه‌رفتن توسط سیستم آنالیز حرکت شش دوربینه و صفحه ثبت نیرو ثبت گردیده و مورد بررسی قرار گرفت. برای تحلیل داده‌ها از آزمون آماری ویلکاکسون استفاده شد.  یافته‌ها: میانگین نیروی اعمال‌شده بر اکستانسور مکانیسم زانو توسط بالشتک حمایتی قوس داخلی به‌علاوه گوه خارجی کاهش معناداری یافت (۰/005=P)، اما با استفاده از دو ارتز دیگر تغییر آماری معناداری به وجود نیامد. نتیجه‌گیری: طبق نتایج این مطالعه، بالشتک حمایتی داخلی به همراه گوه خارجی قادر است بیومکانیک تغییریافته پا در اثر پروناسیون بیش از حد مفصل ساب تالار را نسبت به دو ارتز دیگر بیشتر تصحیح کند. بنابراین می‌توان نتیجه گرفت که استفاده از این ارتز ممکن است در طولانی مدت بتواند با تصحیح راستای نیروها و بازگرداندن پا به ساختار طبیعی، استرس اعمال شده بر بافت‌های ناحیه جلوی زانو را کاهش داده و از عوارض پروناسیون بیش از حد بکاهد.

Title: بررسی تأثیر عوامل شغلی و روانی در کمردرد پرستاران شهر آمل

http://rehabilitationj.uswr.ac.ir/article-1-455-fa.html

هدف: هدف این تحقیق شناخت عوامل شغلی و روانی شغلی با کمردرد پرستاران شهر آمل است. روش بررسی: این مطالعه تحلیلی مقطعی بر روی 400 نمونه به روش سرشماری از کادر پرستاری بیمارستان‌های دولتی شهر آمل انجام شد. جمع آوری اطلاعات با پرسشنامه تغییر یافته نوردیک و تجزیه و تحلیل داده‌ها با آمار توصیفی، مجذور کای، آزمون تی مستقل و رگرسیون لجستیک صورت گرفت. یافته‌ها: 324 نفر (٪81) نمونه‌ها سال گذشته حداقل یک بار کمردرد داشته‌اند. میانگین سنی آنها 6/2±32/39 سال، میانگین قد 8/7±166/7 سانتی‌متر و میانگین وزن 9/8±67/7 کیلوگرم بود. مؤنث بودن (3/03=OR)، ورزش منظم (۰/4=OR)، افزایش قد به ازای هر سانتی‌متر (1/06=OR) و افزایش وزن به ازای هر کیلوگرم (1/04=OR) ارتباط معناداری با کمر درد نشان دادند. خم شدن جهت بلند کردن اشیا از کف اتاق احتمال بروز کمردرد را ٪5 افزایش می‌دهد و از میان عوامل روانی اجتماعی مرتبط با کار به ازای هر یک درجه افزایش در شدت نارضایتی ارتباط با همکاران احتمال بروز کمردرد ٪70 افزایش می‌یابد و از میان عوامل استرس‌زای شغلی، به ازای هر یک درجه افزایش در طیف (همیشه، بیشتر اوقات، بعضی وقت‌ها، هرگز) عجله در کار احتمال بروز کمردرد ٪40 کمتر می‌شود. نتیجه‌گیری: یافته‌های پژوهش تأیید می‌کنند که پرستاران در معرض خطر آسیب‌های کمری هستند. معنادار بودن برخی عوامل فردی، فیزیکی و روانی شغلی با کمردرد نشان‌گر پیچیدگی عوامل مؤثر بر کمردرد است. تعدیل آنها هزینه‌های وارد بر سیستم و فرد را کاهش می‌دهد.
